# T-cell Acute Lymphoblastic Leukemia Presenting With Bilateral Breast Masses

**DOI:** 10.7759/cureus.103002

**Published:** 2026-02-04

**Authors:** Hala Alsoukhni, Shirin Al-Mharat, Ishraq Abu Darweesh, Amani Alrousan, Maher Bani Essa

**Affiliations:** 1 Laboratory Department, Military Cancer Center, Amman, JOR; 2 Radiology Department, King Talal Military Hospital, Mafraq, JOR; 3 Histopathology Department, Princess Iman Center for Research and Laboratory, Amman, JOR; 4 Hematology Department, Military Cancer Center, Amman, JOR

**Keywords:** acute lymphoblastic leukemia, breast, cancer, lymphoma, t-cell

## Abstract

T-cell lymphoma involvement of the breast is rare and represents a diagnostic challenge. Here, we present a case of T-cell acute lymphoblastic leukemia (ALL) in a 20-year-old woman who presented in September 2023 with a history of bilateral breast enlargement associated with general weakness, weight loss, and a feeling of heat, which had lasted for about two months. She was examined clinically and found to have huge bilateral breast enlargement. A breast ultrasound (US) was performed and showed a diffuse abnormal heterogeneous parenchymal appearance of both breasts with multiple enlarged bilateral axillary lymph nodes. The patient was referred to the Military Cancer Center for breast core biopsy, which showed bilateral breast involvement by T-cell lymphoblastic lymphoma. Subsequently, bone marrow examination revealed T-cell ALL. The patient was started on the augmented Berlin-Frankfurt-Münster protocol, achieved complete remission, and maintained it for two years after diagnosis. ALL in the breast is rare but should be considered especially in young patients presenting with sudden breast masses.

## Introduction

Acute lymphoblastic leukemia (ALL) is a hematologic malignancy characterized by the uncontrolled proliferation of immature lymphoid precursors of B or T lineages. It is primarily a disease of children, and most cases are of the B-cell lineage, while T-cell ALL accounts for 15% of pediatric cases and 25% of adult cases. The term lymphoma is used when the neoplastic lymphoblast is restricted to a mass without involving peripheral blood and bone marrow. T-lymphoblastic lymphoma (T-LBL) accounts for 85-90% of all LBL and frequently presents with a bulky mediastinal (thymic) mass. It can occur in any age group but most frequently affects adolescent male patients [1,2].

Primary non-Hodgkin lymphoma of the breast is rare, comprising less than 0.5% of breast malignancy. The majority of breast lymphomas are of the B-cell phenotype, with rare cases of T-cell lineage. Few cases of primary breast T-cell lymphomas have been reported in the literature, including lymphoblastic lymphoma, peripheral T-cell lymphoma (PTCL), anaplastic large cell lymphoma (ALCL), adult T-cell lymphoma, and mycosis fungoides [3].

Usually, low-grade breast lymphomas present as palpable, painless mass similar to that of breast carcinoma, while high-grade lymphomas present as diffuse breast enlargement [4].

Mammography is of limited use in young patients due to high breast density, which reduces mammographic sensitivity. While ultrasound (US) findings are non-specific and variable, it is considered the first-line imaging in young women. The imaging findings need to be accompanied by a tissue biopsy for a definite diagnosis [5].

## Case presentation

A 20-year-old female patient, previously healthy, presented with a history of bilateral breast enlargement that was more on the right breast with pain, general weakness, weight loss, and feeling of hotness for the last two months.

Investigations

Physical examination revealed bilateral generalized breast huge enlargement with tenderness and bilateral axillary lymph node enlargement (LNE). Laboratory investigations were performed, including complete blood count (CBC), which revealed leukocytosis and thrombocytosis. Kidney function tests were within normal ranges. Lactate dehydrogenase (LDH) and uric acid were elevated (Table 1).

**Table 1:** Laboratory results with normal ranges. WBC: white blood cell; LDH: lactate dehydrogenase.

Test	Result	Normal range
WBC	12.4 x 10^3^/µL	4-11 x 10^3^/µL
Hemoglobin	13.2 g/dL	11.5-15.5 g/dL
Platelets	471 x 10^3^/µL	150-450 x 10^3^/µL
LDH	414 U/L	100-248 U/L
Uric acid	7 mg/dL	2.7-6.1 mg/dL

**Figure 1:**
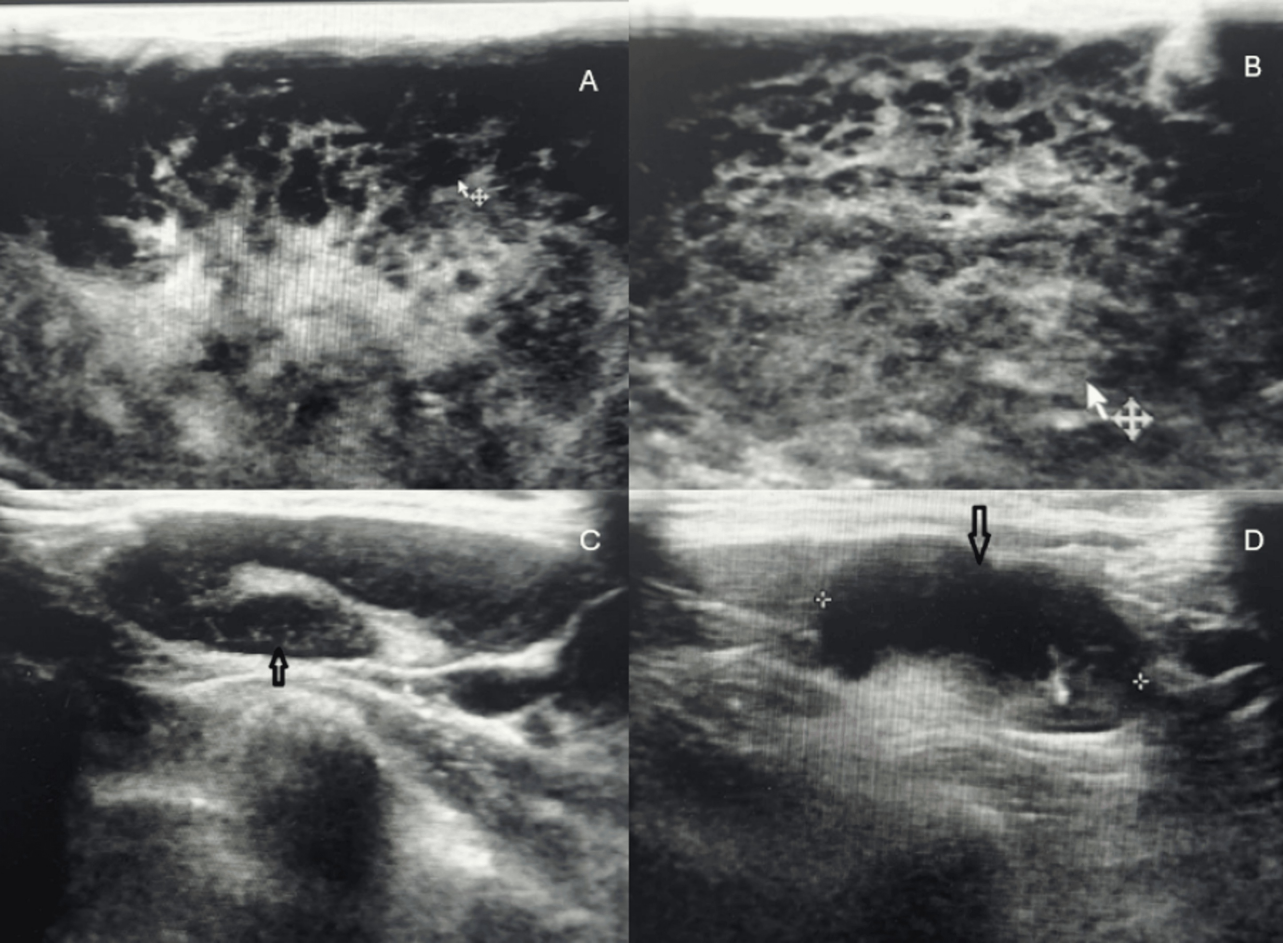
Breast US showing bilateral heterogenous masses (A, B) and enlarged axillary LN (C, D). US: ultrasound; LN: lymph nodes.

Breast ultrasound was performed before referral to our center and showed diffuse abnormal heterogenous parenchymal lesion involving both breasts and diffuse skin thickening with subcutaneous edema and bilateral multiple axillary LNE and categorized as Breast Imaging Reporting and Data System (BI-RADS) 4C (Figure 1).

Diagnosis

**Figure 2:**
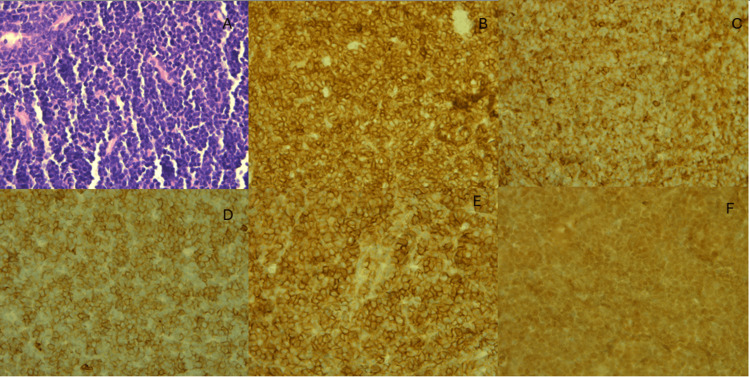
Breast core biopsy. H&E showing diffuse monotonous medium-sized lymphoid cells infiltrating breast tissue (A). Immunostains for CD3 (B), CD4 (C), CD8 (D), CD99 (E), and TdT (F) are positive in these cells. H&E: hematoxylin and eosin stain; CD: cluster of differentiation; TdT: terminal deoxynucleotidyl transferase.

Bilateral breast biopsy was taken, and the microscopic examination revealed medium-sized primitive cells with hyperchromatic nuclei, inconspicuous nucleoli, and scant cytoplasm. Immunohistochemical staining showed positivity for CD3, CD4, and CD8, CD99, and TdT (Figure 2).

**Figure 3:**
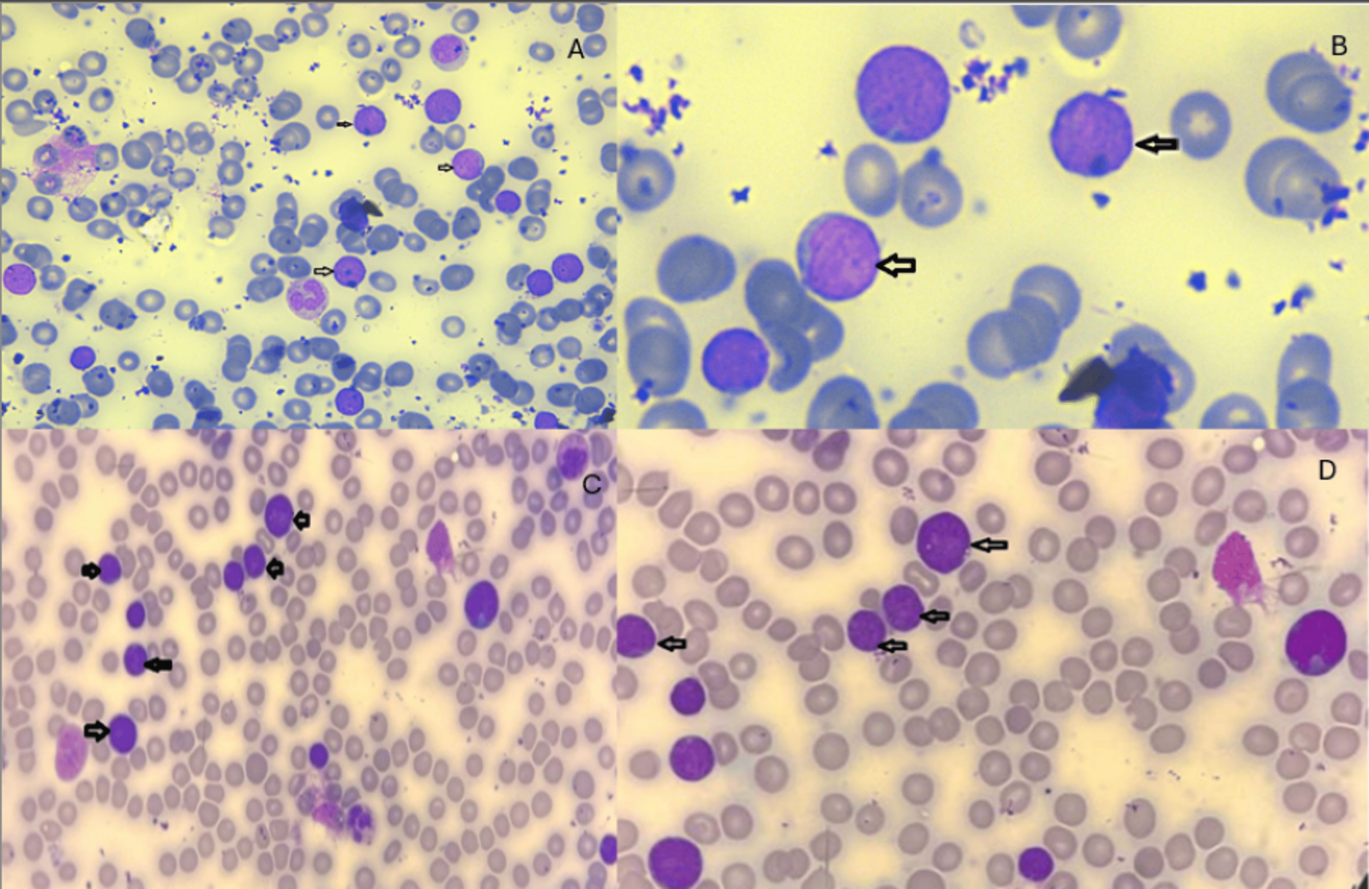
Blasts present in the peripheral blood (A, B) and in the bone marrow aspirate (C, D).

According to these findings, a diagnosis of T-cell lymphoblastic lymphoma (T-LBL) was made, and bone marrow examination was advised to rule out leukemia. Bone marrow aspirate was obtained and showed hypercellular particle with 70% blasts medium in size with high N/C ratio and scant cytoplasm (Figure 3).

**Figure 4:**
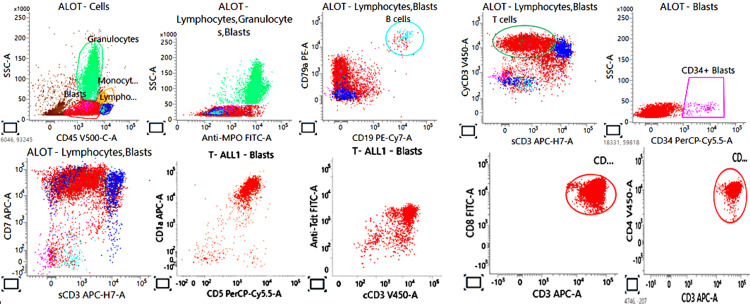
Immunophenotype of the blasts by flow cytometry showing positive cCD3, CD7, CD1a, TdT, CD8, and CD4. TdT: terminal deoxynucleotidyl transferase.

Flow cytometry was performed on bone marrow aspirate specimen and showed positivity of the blasts for the following markers: cCD3, CD4, CD7, CD8, CD1a, TdT, and CD99 (Figure 4).

**Figure 5:**
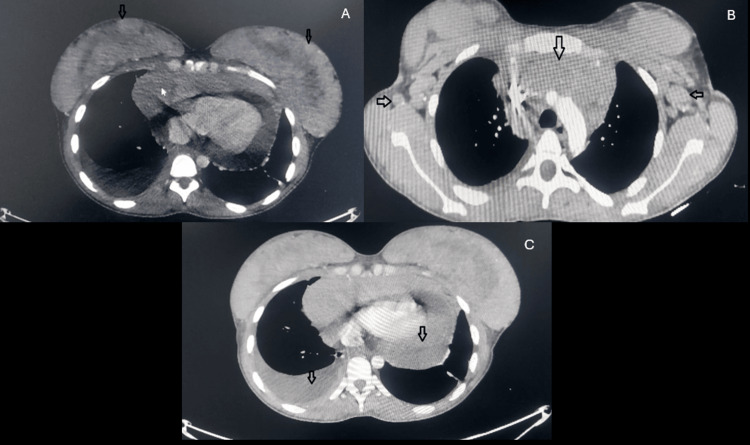
CT scan showing bilateral breast enlargement (A), mediastinal mass and bilateral axillary LNE (B), and pleural and pericardial effusion (C). CT: computed tomography; LNE: lymph node enlargement.

So, the final diagnosis was T-cell acute lymphoblastic leukemia (T-ALL). Lumbar puncture for cerebrospinal fluid (CSF) cytology was performed and revealed no malignant cells. Chest and abdomen CT scans were performed and showed a large mediastinal mass (14.3 cm) with right-sided pleural effusion and pericardial effusion as well as multiple bilateral breast soft tissue lesions with multiple bilateral axillary LNE (Figure 5).

**Figure 6:**
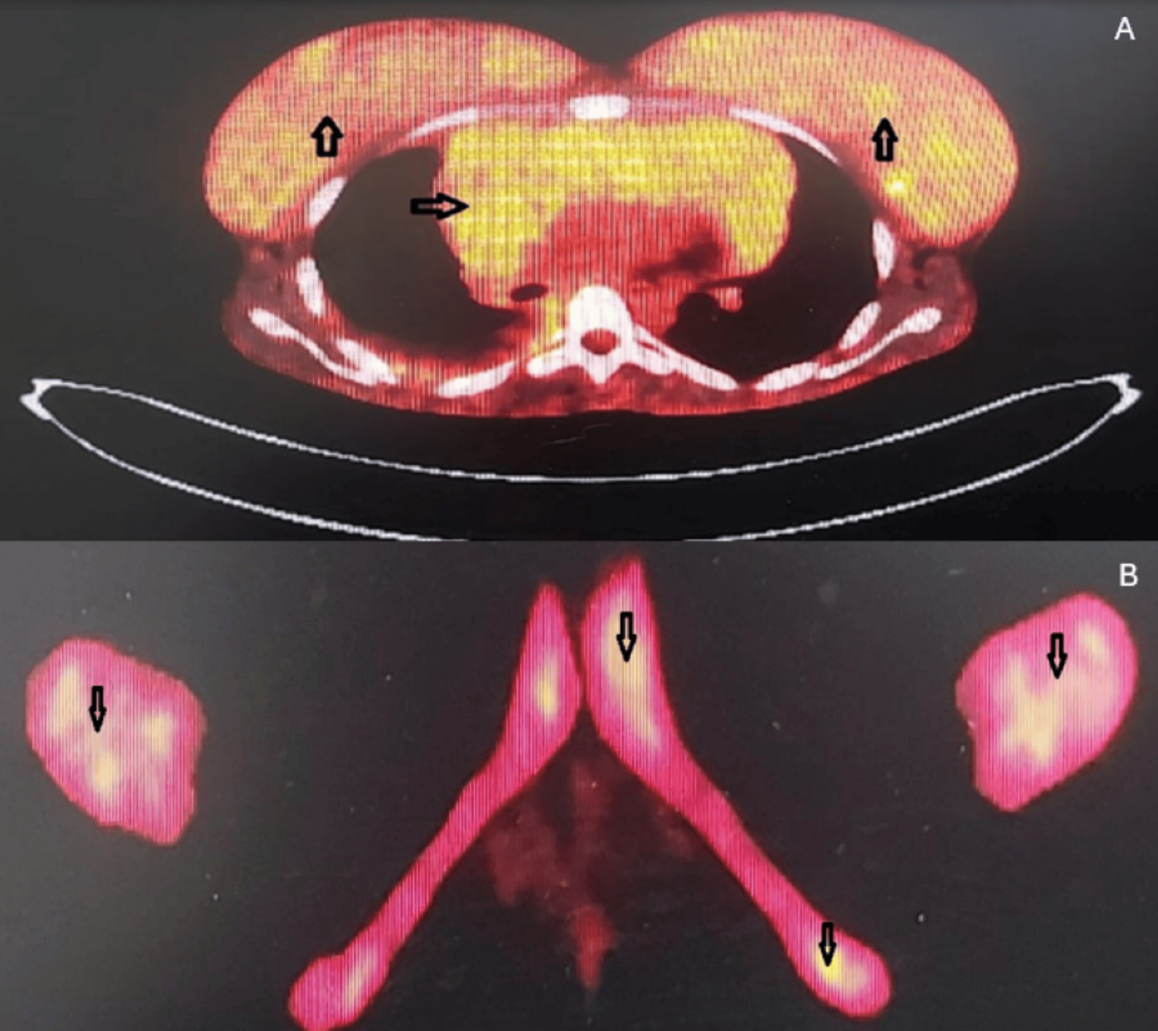
PET scan showing increased radiotracer uptake in both breasts and the mediastinal mass (A) and in the axial skeleton (B). PET: Positron emission tomography

A PET scan was requested and showed a hypermetabolic anterior mediastinal mass starting from the thoracic inlet extending downward to surround the heart (SUV max 8.7) and hypermetabolic left supraclavicular lymph node (SUV max 10.9), bilateral breast hypermetabolic masses (SUV max 6.9), multiple moderately hypermetabolic enlarged bilateral axillary lymph nodes (SUV max 4.5), bilateral pleural effusion more on the right side, and increased activity in the axial skeleton representing bone marrow infiltration (Figure 6).

Treatment

The patient was started on augmented Berlin-Frankfurt-Münster protocol (aBMF), as summarized in Table 2.

**Table 2:** Summary of aBFM treatment protocol. aBFM: augmented Berlin-Frankfurt-Münster.

Phase	Duration	Regimen details
Induction	5 weeks	Adriamycin 25 mg/m²/day IV D1, 8, 15, 28 Oncovin 2 mg IV push D1, 8, day 15, day 28 Prednisolone 60 mg/m²/day D1-28 L-asparaginase 6000 IU/m²/day IM D1, 3, 6, 9, 11, 14, 17, 19, 21 CNS prophylaxis: intrathecal cytarabine 100 mg, methotrexate 12 mg D14
Consolidation	9 weeks	Cyclophosphamide 1000 mg/m²/day IV D0, 28 6-mercaptopurine 60 mg/m²/day PO D0-13, 28-41 Cytarabine 75 mg/m²/day SQ D1-4, 8-11, 29-32, 36-39 Vincristine 1.5 mg/m² /day IV D14, 21, 42, 49 L-asparaginase 6000 IU/m²/day IM D14, 16, 18, 21, 23, 42 Intrathecal methotrexate 12 mg/day D1, 8, 15,22
Interim maintenance	8 weeks	Vincristine 1.5 mg/m²/day IV D0, 10, 20, 30, 40 Methotrexate 100 mg/m²/day IV D0, 10, 20, 30, 40 (escalated by 50 mg/m²/dose) L-asparaginase 15000 IU/m²/day IM D1, 11, 21, 31, 41 Doxorubicin 25 mg/m²/day IV D0, 7, 14
Delayed intensification part 2 reconsolidation	4 weeks	Cytoxan 1000 mg/m²/day IV D28 6-thioguanine 60 mg/m²/day PO D28-41 Cytarabine 75 mg/m²/day SQ D29-32, 36-39 Vincristine 1.5 mg/m²/day IV D42, 49 L-asparaginase 6000 IU/m²/day IM D42, 44, 46, 49, 51, 53 Methotrexate 12 mg IT D29, 36 Repeated for seven cycles
After completion of all cycles, the patient started on POMP protocol since September 2024		6-mercaptopurine 50 mg/m²/day PO D1-28 Prednisolone 40 mg/m²/day D1-5 Vincristine 2 mg/m²/day IV D1 Methotrexate 11 tablets/day D1, 8, 15, 22

Follow-up

**Figure 7:**
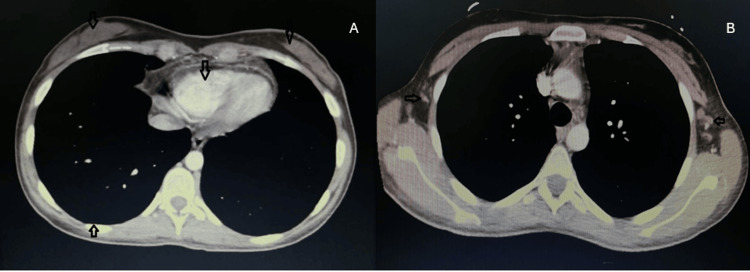
CT scan at three months post treatment showing complete resolution of bilateral breast soft tissue masses and pericardial and pleural effusion and almost complete resolution of mediastinal mass (A) and decrease in the size and number of axillary LN (B). LN: lymph nodes.

A post-induction bone marrow examination was obtained after three weeks and revealed complete remission. Chest and abdomen CT scans were repeated after three months of starting treatment and showed complete resolution of breast masses, complete resolution of pericardial and pleural effusions, almost complete resolution of mediastinal mass, and significant decrease in the number and size of axillary lymph nodes (Figure 7).

CSF cytology was performed regularly before and during chemotherapy, and all results were negative for blasts (Table 3).

**Table 3:** Cerebrospinal fluid cytology results.

Date	Result	Final interpretation
At diagnosis (pre-treatment)		
September 13, 2023	WBCs: 3/cm³ RBCs: 3/cm³	Negative for blasts
After starting treatment		
October 5, 2023	WBCs: Nil RBCs: 3/cm³	Negative for blasts
October 17, 2023	WBCs: 3/cm³ RBCs: Nil	Negative for blasts
October 24, 2023	WBCs: Nil RBCs: Nil	Negative for blasts
November 1, 2023	WBCs: 3/cm³ RBCs: 145/cm³	Negative for blasts
November 8, 2023	WBCs: 3/cm³ RBCs: 133/cm³	Negative for blasts
April 15, 2024	WBCs: Nil RBCs: 13/cm³	Negative for blasts
April 18, 2024	WBCs: Nil RBCs: 13/cm³	Negative for blasts
July 14, 2024	WBCs: 3/cm³ RBCs: 3/cm³	Negative for blasts

The patient is under ongoing follow-up in the clinic, her last visit was on January 2026, she is on maintenance chemotherapy POMP protocol (Prednisolone, Oncovin, Methotrexate, Purinethol), and she is clinically doing well.

## Discussion

T-cell ALL accounts for around 15% of childhood ALL and 25% of adult ALL and typically presents with a high leukocyte count, lymphadenopathy, and an anterior mediastinal mass with acute respiratory symptoms. T-cell LBL frequently presents as a rapidly growing anterior mediastinal mass with pleural effusion [1]. Breast involvement is a rare primary presentation [6]. Leukemic infiltration of the breast is unusual and mostly occurs in acute myeloid leukemia (AML) [7,8]. Breast involvement in leukemia can occur in three different situations. First, it can occur before bone marrow involvement; second, it can occur as systemic disease at the time of leukemia presentation; and third, it can present as a relapse after treatment of acute leukemia [9]. Leukemia in the breast is usually bilateral as it is not a local disease [10]. Other sites of involvement include the central nervous system (CNS), spleen, liver, tonsils, skin, and gonads [1,11]. LBL and ALL are clearly related, LNE dominates in LBL, and blasts in the BM dominates in ALL. Lymphoblasts have a more immature thymic phenotype in ALL,whereas in LBL T-cells are more mature, but it is actually difficult to differentiate de novo ALL from the leukemic transformation of LBL [12].

Breast lymphomas comprise a small percentage of breast malignancies (0.1%-0.5%) and usually present with unilateral breast involvement, more commonly on the right side, and in 1-14% of cases involve both breasts, frequently presenting with incidental breast nodules or ipsilateral axillary lymphadenopathy as initial symptoms. Other manifestations include local skin erythema, and nipple discharge. There is no significant difference in the clinical presentations of breast lymphoma and breast cancer [13].

In our patient, the presentation was bilateral breast enlargement, which lasted for about two months, followed by weight loss and general weakness.

The mammogram findings in cases of breast involvement by acute leukemia are usually difficult to identify, so mammogram has minimal usefulness in these cases [14]. Ultrasonographic findings are not fully prescribed and may vary, but it is considered the best imaging technique [12,15]. In our case, the patient underwent ultrasound imaging, which showed a diffuse heterogeneous parenchymal appearance with ill-defined lesions, and a PET scan, which revealed hypermetabolic active lymphomatous disease involving multiple LN groups above the diaphragm, an anterior mediastinal mass, bilateral breast masses, and BM infiltration.

The diagnosis is usually made by fine needle aspiration or, preferably, core biopsy with immunohistochemical staining [16,17]. In our patient, bilateral breast biopsies were obtained, and after making final diagnosis of LBL, a bone marrow examination was performed to check for ALL. Treatment of breast lymphoma should be similar to that of other extranodal lymphomas, with chemotherapy and radiotherapy. Surgical treatment is not indicated except as a diagnostic method [13,18].

The rarity of breast LBL and its similarity to breast cancer may delay the diagnosis in such cases, and the clinician should be aware of this entity to avoid misdiagnosis [4]. Precise diagnosis is important to go for proper treatment and avoid unnecessary surgical procedures, as reported in a previous case [19].

Limitations

This case has limitations inherent to its design. The rarity of this case limits comparison with similar reported cases. Also, the nonspecific imaging findings and lack of molecular testing may cause some restriction in the prognostic considerations.

## Conclusions

Breast ALL is rare but should be considered especially in young patients presenting with sudden breast masses. Comprehensive integration of imaging, histopathologic, and immunophenotypic findings is crucial for establishing an accurate diagnosis to ensure proper treatment and avoid unnecessary surgical interventions. Reporting such cases raises awareness of this uncommon presentation and minimizes the possibility of delays in diagnosis and misdiagnosis.
